# Extralobar pulmonary sequestration with elevated serum neuron-specific enolase: A case report and review of the literature

**DOI:** 10.1097/MD.0000000000022574

**Published:** 2020-10-02

**Authors:** Feng-Wei Kong, Wei-Min Wang, Longbo Gong, Wenbin Wu, Miao Zhang

**Affiliations:** a Department of General Surgery, Xuzhou Infectious Disease Hospital; b Department of Surgery, Xuzhou Central Hospital, Xuzhou, China.

**Keywords:** pulmonary sequestration, three-dimensional computed tomography angiography, video-assisted thoracoscopic surgery

## Abstract

**Rationale:**

Pulmonary sequestration (PS) presenting with elevated serum tumor markers is rare, and it might be misdiagnosed as malignancy.

**Patient concerns:**

A 26-year-old asymptomatic male patient was admitted because the x-ray showed an intrathoracic lesion. Meanwhile, the serum neuron-specific enolase (NSE) was elevated. Three-dimensional computed tomography angiography revealed an isolated feeding vessel arising from the aorta.

**Diagnoses:**

Extralobular PS was confirmed by computed tomography angiography and postoperative pathological staining.

**Interventions:**

Two-port thoracoscopic resection of the sequestrated lobe was performed.

**Outcomes:**

The serum NSE decreased to within the normal range and persisted during the follow up of 10 months.

**Lessons:**

A thorough work-up should be considered for the PS patients presenting with abnormal serum NSE. Detailed knowledge regarding the relationship between NSE and PS necessitates further studies.

## 1 Introduction

Pulmonary sequestration (PS) is defined as a non-functioning lung tissue that lacks a normal connection with the tracheobronchial tree and has an unusual feeding artery from the aorta. About 91.3% of the PS patients have an aberrant arterial supply from the descending thoracic aorta without accompanying bronchus.^[1]^ Contrast-enhanced computed tomography (CT) or magnetic resonance imaging (MRI) is the main technique for the diagnosis of PS; moreover, a timely surgical resection of the sequestrated lobe by is the major treatment of choice.^[2]^ To our knowledge, PS presenting with elevated tumor biomarkers is uncommon.

Neuron-specific enolase (NSE) can be of value in the diagnosis of small cell lung cancer, neuroendocrine tumors, all stages of neuroblastoma, melanoma, seminoma, renal cell carcinoma, Merkel cell tumor, carcinoid tumors, dysgerminomas and immature teratomas, malignant pheochromocytoma, Guillain-Barré syndrome, and Creutzfeldt-Jakob disease.^[3]^ In addition, NSE might be utilized as a prognostic and therapeutic biomarker for neuroinflammation, neurodegeneration, and neuroprotection in spinal cord injury as well as neurodegenerative diseases.^[4]^

However, the relationship between NSE and PS has not been elucidated. Herein we presented a case of extralobular PS with elevated serum NSE before surgery, followed by a brief review of the relevant literature.

## 2 Case presentation

**Figure 1:**
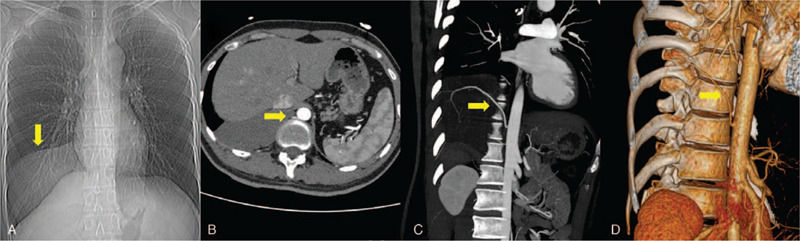
The images of the intrathoracic lesion (indicated by arrows). A. The x-ray in August 2019 showed an intrathoracic lesion. B. The blood supply of the lesion from the adjacent descending thoracic aorta. C and D. Preoperative 3D-CTA confirmed the feeding vessel of the sequestrated lobe.

This report was approved by the Institutional Review Board of Xuzhou Central Hospital. Written informed consent was obtained from the patient. The clinical data were presented anonymously for privacy concerns. A 26-year-old asymptomatic male non-smoker was admitted to the local hospital in August 2019 because his chest X-ray revealed an intrathoracic shadow about 12.5 cm (Fig. 1A). His previous medical history was unremarkable, and the physical examination showed nothing abnormal. However, the laboratory tests indicated normal carcinoembryonic antigen (CEA), β-human chorionic gonadotropin, alpha-fetoprotein, squamous cell carcinoma antigen, carbohydrate antigen (CA) 72-4, CA125, CA19-9, and cytokeratin-19 fragment (CYFRA 21-1); whereas the NSE was slightly increased (16.5 ng/mL; normal range < 13 ng/mL). Further CT revealed a mass located in the mediastinum (Fig. 1B). Three-dimensional CT angiography (3D-CTA) was utilized,^[5]^ which demonstrated an anomalous artery arising from the descending thoracic aorta (C and D).

Based on these findings, a right extralobular PS was diagnosed. His cranial MRI, abdomen CT and emission CT excluded other detectable malignancies. Two-port video-assisted thoracoscopic surgery (VATS) resection of the sequestrated lobe along the feeding vessel was scheduled after a multidisciplinary evaluation.

Resection of the sequestrated lung was performed under general anesthesia. The operation time was 60 minutes, and the estimated blood loss was 100 mL. Ultrasound-guided serratus anterior plane block was utilized for analgesia. Postoperative anatomy of the specimen confirmed the presence of an aberrant feeding artery, while the findings from pathological staining were consistent with PS. Further immunohistochemistry tests of the sequestrated lobe indicated positive expression of neuron-specific enolase and negative expression of S-100, chromogranin A and synuclein. The patient displayed an uneventful course after the operation. His serum NSE levels decreased to within the normal range a month after the surgery. Local or distant malignant lesions were undetectable, and the normalized serum NSE persisted during the follow up of 10 months.

## 3 Discussion

NSE is a nonspecific biomarker for cancer. The present case confirms that elevated serum NSE may be found in PS. After resection of the sequestrated lobe, his serum NSE level was normalized. No disease of the digestive or respiratory system was detected during the follow up. Therefore, it is possible that the elevated NSE was caused by the PS. Nevertheless, the occasional finding of abnormal NSE level in the patient should be interpreted with caution to avoid potential diagnostic pitfalls. Furthermore, the underlying mechanisms are unknown. A hypothesis is not appropriate due to our limited knowledge; thus, an analysis of the relationship between NSE and PS is not conducted to avoid misleading information. Future researches are warranted to address this issue.

We searched PubMed, Web of Science, Scopus, Embase, and Google Scholar from their inception to May 2020 for reports of PS presented with abnormal serum tumor markers. Finally, a total of 19 PS cases with elevated tumor markers were obtained. The clinical features were summarized in Table 1. Among these reports, the elevated biomarkers included CA 19-9 (14 cases), CA 125 (5 cases), CEA (5 cases), CA 50 (1 case), CYFRA 21-1 (1 case), NSE (1 case), NCC-ST-439 (1 case), free normetanephrine (1 case), and sialyl Lewis X-i (SLX; 3 cases), which were all decreased to within the normal range after resection of the sequestrated lung tissues, regardless of the concomitant ovarian cyst, bronchogenic cyst or mycosis.

**Table 1:**
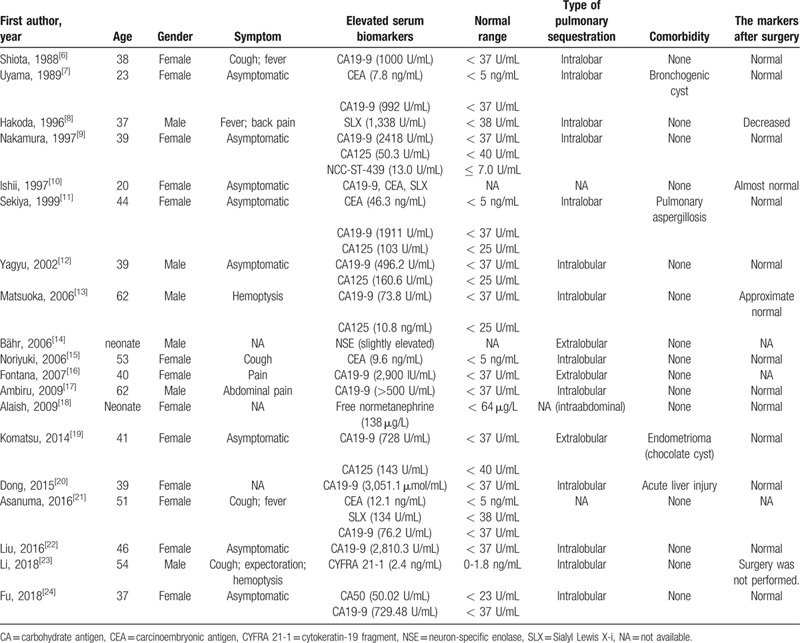
Previous reports of pulmonary sequestration with elevated tumor markers (a total of 19 cases).

The normetanephrine and metanephrine are useful in the screening for pheochromocytomas.^[25]^ Moreover, the plasma-free normetanephrine is helpful in the diagnosis of heochromocytomas and paragangliomas;^[26]^ whereas the carbohydrate antigen NCC-ST-439 has been proved to be a biomarker for Dukes’ C colorectal carcinoma and breast cancer.[^27^,^28^] Furthermore, SLX indicates the potential metastasis and/or extension of carcinoma.^[29]^ In addition, the change of serum and urine CYFRA 21-1 is efficient for the diagnosis of bladder cancer.^[30]^ Besides CEA, CYFRA 21-1 provides diagnostic, therapeutic, and prognostic information for non-small cell lung cancer patients.^[31]^ On the other hand, several non-neoplastic conditions including inflammations, benign tumors, renal or hepatic insufficiency are also associated with elevated plasma CEA,^[32]^ which is a biomarker in colorectal cancer for diagnosis and monitoring of response to therapy.^[33]^ CA 125 is a biomarker of ovarian cancer;^[34]^ whereas CA 19-9 is a validated marker for the diagnosis of pancreatic cancer.^[35]^ However, the elevated CA 50 can be observed in gastrointestinal cancers as well as the malignancies outside the digestive tract.^[36]^ It is reported that the increased CA 19-9 and CEA in bronchogenic cyst, intestinal duplication and PS can be explained for the common embryogenic origin of respiratory and digestive apparatus,^[16]^ but a definite conclusion regarding the role of NSE in PS could not be drawn from the current studies. To date, there are no reliable biomarkers for the differential diagnosis of malignancies and PS; therefore, radiographical tests and blood surveillance are necessary after the resection of PS when the patients present with elevated tumor biomarkers.

In summary, a thorough work-up is useful during the diagnosis of PS with abnormal serum NSE, and strict surveillance of the changed biomarkers is necessary before it decreases to within their normal range. However, further studies regarding the underlying mechanisms are needed to verify this occasional finding.

## Author contributions

**Conceptualization:** Feng-Wei Kong, Wei-Min Wang

**Data curation:** Wei-Min Wang

**Funding acquisition:** Wenbin Wu

**Methodology:** Longbo Gong

**Resources:** Miao Zhang

**Writing – original draft:** Feng-Wei Kong, Wei-Min Wang, Wenbin Wu

**Writing – review & editing:** Miao Zhang, Wenbin Wu
